# Anger and psychological symptoms relationship: mediator role of maladaptive schemas

**DOI:** 10.3389/fpsyg.2023.1183618

**Published:** 2023-07-17

**Authors:** Ahmet Özmen

**Affiliations:** Department of Guidance and Psychological Counseling, Faculty of Education, Kafkas University, Kars, Türkiye

**Keywords:** trait anger, psychological symptoms, early maladaptive schemas, schema therapy, mental health

## Abstract

Trait anger is the strong predictor of various psychological symptoms like anxiety, depression, and hostility. Explaining how and why this relationship occurs is crucial to come up with more effective prevention and intervention strategies in the field. To this end, the current study aimed to reveal the mediating effect of early maladaptive schemas, which is the basic concept of schema therapy, on the relations of trait anger and psychological symptoms. Data was collected from 301 university students by using the measurement tools of Brief Symptom Inventory, Trait Anger Scale and Young Schema Scale. Findings revealed that trait anger positively predicted psychological symptoms. Secondly, a set of predictive models were prepared to detect the mediating effect of early maladaptive schemas. According to the analysis in the last stage, early maladaptive schemas fully mediated the relationship between trait anger and psychological symptoms.

## 1. Introduction

Early maladaptive schemas (EMSs) are usually formed by the influence of early memories, which may be related to the individual’s self and relationships with others. The emotions, cognitions, and bodily sensations that emerge under the influence of these memories constitute the concept of EMSs. They start to form at an early age and continues recurrently throughout one’s lifetime. These schemas are also defined as destructive, emotional, and cognitive patterns (Young et al., 2003; Thimm, 2010c). In accordance with the explanations of schema therapy, EMS formation occurs in childhood when developmental needs are inadequately met in a normal and healthy way during this period. The innate temperament also has an effect on the formation of these structures. They are psychological constructs that individuals believe to be unconditionally true for them, for the world, and for others (Bach et al., 2018; Farrel and Shaw, 2018). Through EMSs, individuals justify themselves to survive. Hence, schemas are resistant to change. Individuals perceive schemas as *a priori* truths; therefore, these schemas also influence their later-life experiences. These schemas influence their thoughts, feelings, behaviors, and relationships with others. Paradoxically, they cause individuals to recreate conditions in adulthood that hurt them, mostly in childhood (Young et al., 2003).

On the other hand, functional schemas improve when the child’s essential needs are adequately met, which leads the child to gain positive perceptions of themselves, others, and the world as a whole (Arntz and van Genderen, 2009). Thus, childhood interactions bring about the formation of EMSs, which play decisive roles within the susceptibility to various forms of psychopathology (Harris and Curtin, 2002; Bach et al., 2018). EMSs, which are often implicit, become especially active through life experiences and psychopathologies. In other words, they are automatically activated when a relevant situation is encountered (Pretzer and Beck, 2004; Bagdacicek, 2009; Haugh et al., 2017).

Previous studies on EMSs have shown that EMSs are related to psychological problems or play mediating roles (Ball, 2007; Riso et al., 2007; Sheffield et al., 2009; Shute et al., 2019). To illustrate, research in this area suggests that psychological maltreatment in childhood is related to depressive symptoms at older ages (Paul and Eckenrode, 2015). Moreover, Cámara and Calvete (2012) demonstrated the relationship between EMSs, depression, and anxiety. Additionally, emotional maltreatment and emotional carelessness correlated with future anxiety and depression, and EMSs had a mediator role in this relationship (Wright et al., 2009). Some maladaptive schema domains have a mediatory function in the relationship between attachment style and depression in youth (Roelofs et al., 2011). Moreover, the relationship between some schema domains and the severity of depressive symptoms seem to be closely linked. These schema domains predict treatment outcomes and drastical changes during the treatment process (Renner et al., 2012). A similar study indicated that changes in EMSs predict symptomatic relief in individuals with or without personality disorders (Nordahl et al., 2005).

Mediating role of EMSs is also captured in the relationship between perceived parental behavior and personality disorder outcomes (Thimm, 2010b). Similarly, negative family functions predicted psychological symptoms (Kapci and Hamamcı, 2010). Moreover, individuals with borderline personality disorders were found to have higher scores in addiction/inadequacy and imperfection/embarrassment schema domains (Jovev and Jackson, 2004). Thus, different schema domains may be related to various psychopathologies. Furthermore, patients with somatoform disorder had significantly higher EMS scores than those with no such diagnosis (Kırpınar et al., 2014). To this end, general early trauma appears to cause the development of EMSs.

While explaining the psychological symptoms, besides EMSs, role of anger focused emotion regulation attracts the attention. The issue of emotion and emotion regulation has gained importance in psychology in the last decade (Leahy, 2015). Such an emphasis on emotion and emotion regulation has further increased the importance of anger. In addition, anger is a key criterion in five diagnoses within the scope of the *Diagnostic and Statistical Manual of Mental Disorders, Fifth Edition* diagnostic criteria (Fernandez and Johnson, 2016). Anger is a primary emotion that occurs in life-threatening or various stressful situations. In other words, the perception of threat can stimulate anger (Novaco, 2016). Anger is evaluated as an emotional response to unsatisfied wishes, unwanted consequences, and unfulfilled expectations. While it warns the individual of a problem, it informs the person about the likelihood to behave in a harmful or aggressive way (Soykan, 2003; Farrel and Shaw, 2018). Especially, a lack of ability or problems in emotion regulation was suggested to be related to various physical and mental health problems during the early periods of life (Aldao et al., 2010). For instance, studies related to the causes of physical abuse and self-harm behavior at early ages have indicated that individuals harm themselves to control the emotional distress they experience and punish themselves (Aksoy and Ögel, 2003). To illustrate, anger/anger expression styles are connected with psychological symptoms such as depression and anxiety and ruminative personality traits (Conger et al., 2003; Harper and Arias, 2004; Erdur-Baker et al., 2009), eating disorders (Oral and Sahin, 2008), and chronic pain (Sayar et al., 2001; Oz et al., 2011).

In a study considered important in terms of emphasizing the relationship between anger and attention-deficit hyperactivity disorder (ADHD), Ramirez et al. (1997) concluded that people with ADHD had high levels of trait anger, which could complicate their daily lives and negatively affect their school activities. Moreover, Richards et al. (2002) reported that students with ADHD displayed angrier behaviors while driving and were more frequently involved in traffic accidents.

It is evident that anger negatively affects an individual’s psychological and physiological health when it is not expressed appropriately (Bilge, 1992). In that sense, it was emphasized that anger poses serious threats to physical health, such as headaches (Oz et al., 2011), chronic cardiovascular diseases, high blood pressure, and stomach diseases (Soykan, 2003). Considering that anger is related to negative impacts on physiological and psychological health, such as subjective distress and conflicts in personal relationships, interest in anger control prevails as a human welfare and societal concern (Taylor and Novaco, 2005).

Despite the increasing importance of anger, studies on its interaction with EMSs are scarce. Problems with emotion regulation that individuals bring from childhood to the present can lead to the maintenance of their schemas (Eldoğan and Barışkın, 2014). Some schema domains played a mediating role in attachment and anger relationship (Haspolat and Şendağ, 2018). Saritas-Atalar and Altan-Atalay (2018) asserted that the relationship between maternal rejection and psychological problems such as depression and anxiety is mediated by EMSs. A study on female crime victims showed that anger is associated with the development of post-traumatic stress disorder (PTSD) (Riggs et al., 1992).

## 2. The present study

Based on the previous findings mentioned above, people with high trait anger are more likely to develop psychological symptoms. Rather than just capturing this information, explaining this relationship would enlighten the future research and practices. To this end, EMSs seem to be one of the possible third factors to explain this association. In addition to the link between anger and psychological symptoms, it is understood that EMSs are also associated with both anger and psychological symptoms. However, to the best of our knowledge, the possible explanatory role of EMSs in the relationship between trait anger and psychological symptoms has not yet been clarified. This will be beneficial for both researchers and practitioners in the field of psychology. In this context, the following questions were addressed in this study:

1. Does a significant relationship exist between trait anger and psychological symptoms, and is trait anger a significant predictor of psychological symptoms? (Model 1).
2. Can EMSs predict psychological symptoms, and do they affect the predictive relationship of trait anger and psychological symptoms? (Model 2).
3. What is the mediating role of EMSs in the relationship between trait anger and psychological symptoms? (Model 3).

### 2.1. Research model

In this study, the impact of EMSs on the prediction of psychological symptoms related to the trait anger variable was examined using a mediating variable. The relationships between trait anger, psychological symptoms, and EMSs were explained using an associational model in which mediating role of EMSs was examined between the exogenous variable of anger and indogenous variable of psychological symptoms. This is a descriptive study, as it was conducted by gathering evidence about the validity of the model established (Fraenkel and Wallen, 2009).

## 3. Methods

### 3.1. Participants

To achieve the purpose of this study, the study population included university students, not only they were easier to access as participants, but they are likely to have more insight about themselves compared to uneducated people. Thus, the study group consisted of university students who agreed to voluntarily participate in the study. Before the data collection process, *a priori* power analysis was carried out to determine the required sample size for a relatively moderate effect size (0.20) with 0.80 statistical power and 0.05 probability level for Structural Equation Model (SEM) with 3 latent and 29 observed variables (Soper, 2023). Power analysis suggested to have at least 296 participants. However, considering the possible lost in the data set, extra number of participants were reached out. Initially, 422 participants (Turkish university students) were reached out using a convenient sampling method. First, missing data were screened and excluded from the dataset. These cases were exluded as the related participants did not complete the data collection process by stopping it in the middle of the procedure. After this process, 301 university students remained, of whom 135 (44.9%) were male and 166 (55.1) were female and were included in the study set. Of the university students who participated in the study, 98 (32.6%) were freshmen, 41 (13.6%) were sophomores, 49 (16.3%) were juniors, and 113 (37.5%) were seniors. The participants were between the ages of 17 and 28 years (mean, 20.8 years).

### 3.2. Measures

#### 3.2.1. Brief symptom inventory

This inventory consists of 53 items and was developed in 1992 by Derogatis (1992). Sahin et al. (2002) adapted the scale into Turkish, modifying it to include 5 subscales: Anxiety, Depression, Negative Self, Somatization, and Hostility. As a result of the analyses, the adapted scale had a reliability score of 0.94. The variance explained in the scale with its 5-factor structure was 32% (Sahin and Durak, 1994; Sahin et al., 2002). On the other hand, in this study, according to the exploratory factor analysis, the explained variance rate was 51.4%, and the reliability coefficient was 0.964. The fit index values of the factor analysis (confirmatory) were as follows: *χ*^2^ = 3906.74, *p* < 001, root mean square error of approximation (RMSEA), 0.076; and comparative fit index (CFI)/normed fit index (NFI), 0.96/0.94. Some example items for scale are: “Feeling hopeless for the future” and “Trouble falling asleep.” According to the results, the scale used in this study complied with the adapted scale.

#### 3.2.2. Trait anger-anger expression style scale

The scale used in this study, which consisted of 34 items based on self-expression, was developed by Spielberger (1988). Ozer (1994) adapted it into Turkish. This measuring tool consists of 34 items under the following categories: Trait Anger (10), Anger Control (8), Anger Out (8), and Anger In (8). The reliability test revealed the internal consistency coefficients for trait anger (0.79), anger control (0.84), anger out (0.78), and anger in (0.62). The alpha values of the trait anger scale ranged from 0.67 to 0.92 (Ozer, 1994). Using the same scale, Karababa and Dilmac (2015) concluded that the internal consistency coefficient for the trait anger scale as 0.86. Within the scope of this study, the analyses were based on the participants’ trait anger scores. An example item for this scale is: “Being critized in front of others make me angry.” On the basis of these data, the scale used was found to be valid and reliable.

#### 3.2.3. The young schema scale

The Young Schema Scale consists of 90 items. In this study, the 3-item Young Schema Scale-Short Form, which was developed by Young et al. (1990, 2003) and adapted into Turkish by Soygüt et al. (2009), was used. While the original scale has 18 factors, the adaptation into Turkish consisted of only 14 factors. A higher-order factor analysis was performed on the factors obtained, and five schema domains were identified: Disconnection, Other Directedness, Unrelenting Standards, Impaired Autonomy, and Impaired Limits (Soygüt et al., 2009). Within the scope of this research, factor analyses (exploratory and confirmatory) were applied to the scale. In the factor analysis (exploratory), the explained variance ratio was 52.4%, and the reliability coefficient was 0.955. The fit index values of the confirmatory factor analysis were *χ*^2^ = 5893.1, *p* < 0.001, RMSEA = 0.056, and CFI/NFI = 0.94/0.89. An example item for this measurement tool is: “I worry that people I feel close to will leave me or abonden me.” According to the results, the scale used in this study conforms the adapted scale.

## 4. Procedure and data analyses

### 4.1. Research procedure

After obtaining the ethical approval of from ethics committee of a state university, questionnaire sheet was distributed to the participants by the researcher in paper-pencil format. They participated the study on voluntary base after reading the informed consent form. The scales were implemented in the 2019–2020 academic year. At the end of the study, the participants were debriefed and thanked.

### 4.2. Statistical procedure

In this study, the assumptions to be tested were examined before performing the confirmatory factor analysis. In the first stage, a missing data analysis was performed on each component of the model. In the second stage, the data were converted to *Z* scores, and Mahalanobis distances were calculated to determine the outliers. In the third stage, Shapiro–Wilk scores were calculated to test the normality of the data. The total scores of all subdimensions, except the unrelenting standard subdimension, were found to be normally distributed. When testing for normality, in cases where the number of data were not so large, values with Shapiro–Wilk scores >0.05 were accepted as having a normal distribution (Tabachnick and Fidell, 2007). The last assumption was multicollinearity, which was calculated separately for each variable. To eliminate the multicollinearity problem, the variance inflation factor rate should be <10, and condition index (CI) values should be <30 (Kline, 2005; Buyukozturk, 2010). The analyses conducted for this study revealed no multicollinearity problem in the data.

After the assumptions were tested, the data were found to be in accordance with the multivariate statistics, but some variables failed to meet the assumption of normality. In cases where the data did not meet the expected normality, the robust maximum likelihood technique, which is the most appropriate parametric estimation method, proposed by Jöreskog (2002), was administered. It was used in cases with a normal data distribution.

In testing the hypotheses of the present study, the statistical procedure was determined on the basis of the assumptions of Baron and Kenny (1986). They stated that (1) the independent and dependent variables of the study should be related to each other, (2) in the study, the independent variable should be associated with the mediator variable, (3) the variable investigated for its mediating role should be associated with the dependent variable, and (4) when the impact of the mediator variable on the dependent variable is controlled, the relationship between the independent and dependent variables should significantly decrease. In that sense, a series of models to test these assumptions were formed through the latent variable structural equation modeling using the LISREL 9.2 program. In Model 1, the relationship between trait anger and psychological symptoms was tested. Two more models were then drawn by including EMSs in the predictive models to identify possible mediator roles. In addition, correlation matrices were used in the structural equation models.

## 5. Results

The analyses were completed in three stages for the questions asked in the research process. In the first stage, the predictor relationships between anger and psychological symptoms were determined. In the second stage, the EMSs were included in the analysis to test their mediating role. The models created in this context and the fit index values obtained regarding these models are given.

**Figure 1:**
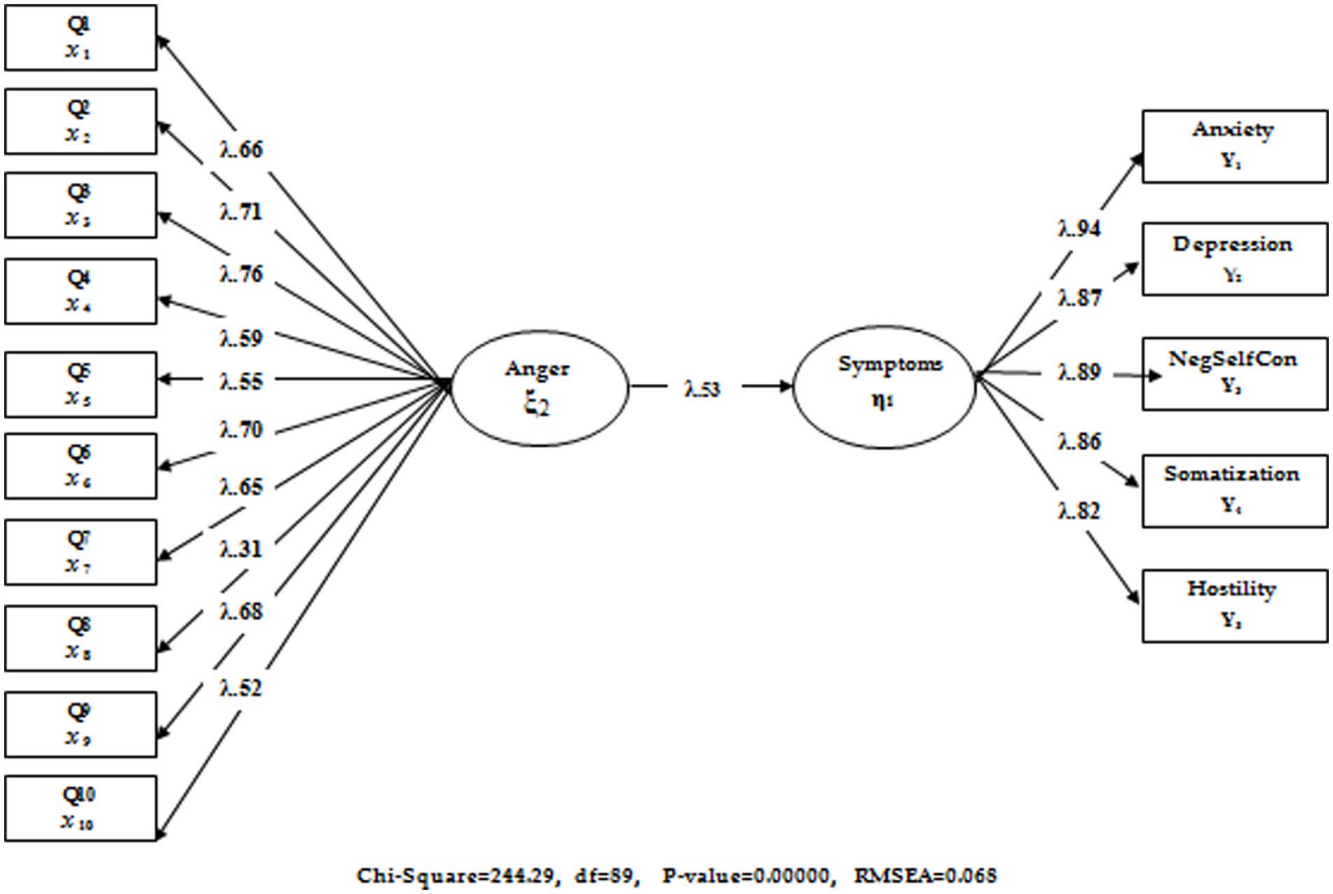
The relationship between trait anger and psychological symptoms.

In the first stage, hypothesis 1, designed as Model 1 and expressed as “Does a significant relationship exist between trait anger and psychological symptoms, and can trait anger significantly predict psychological symptoms?” was tested. In this model, trait anger is expected to predict psychological symptoms positively. The results are illustrated in Figure 1.

**Figure 2:**
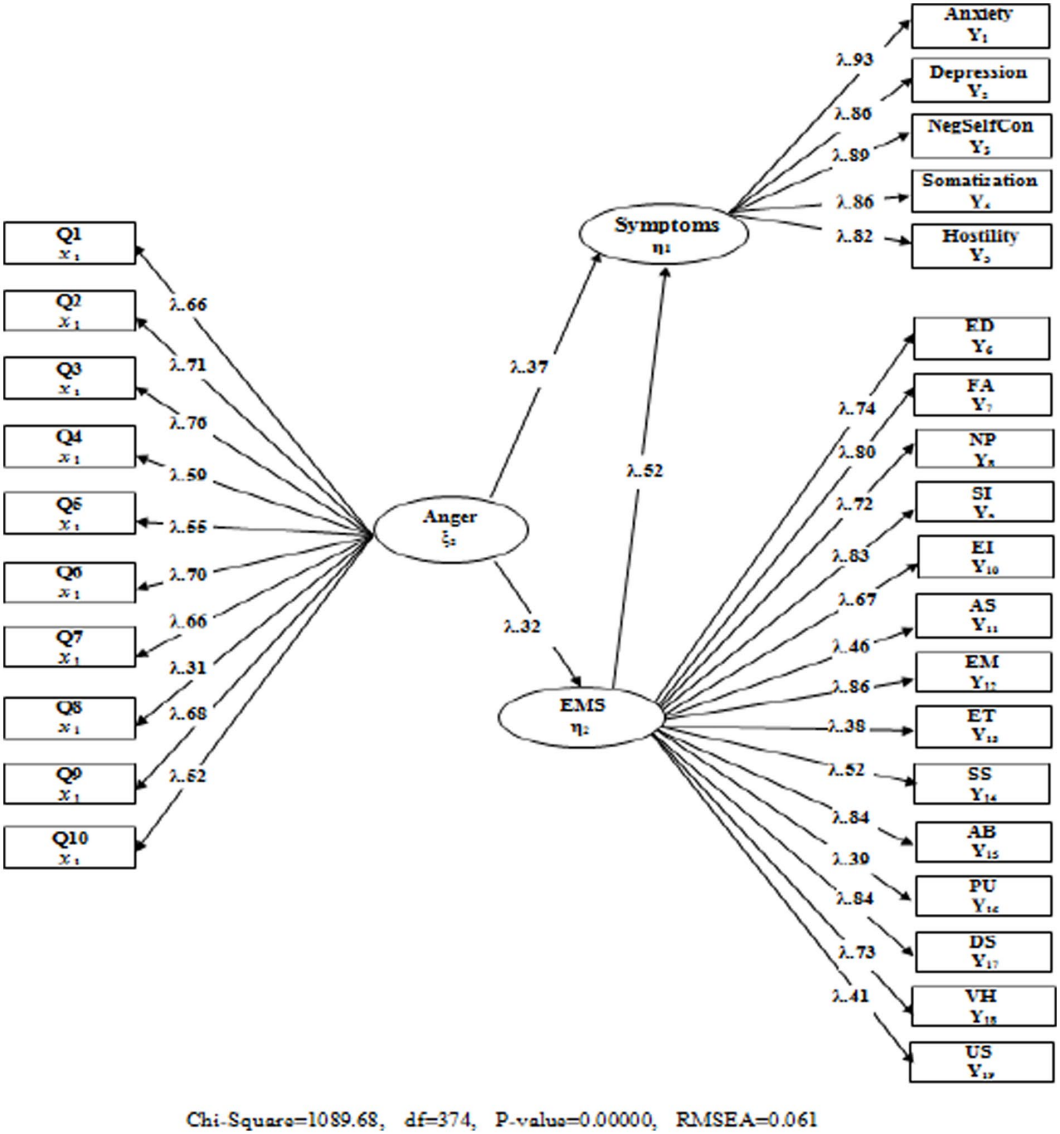
The standardized model including early maladaptive schemas.

When the fit index values for the tested model shown in Figure 1 (*χ*^2^/sd *=* 2.74; CFI = 0.94; TLI = 0.94; RMSEA = 0.068) were considered, from this point, both the latent and observed variables represented a significant relationship (*p* < 0.001). Accordingly, the trait anger variable predicted psychological symptoms positively and significantly (*β* = 0.28, *p* < 0.01). The findings showed that trait anger had a strong relation with psychological symptoms and is an important risk source in this sense. In the second stage, hypothesis 2 as Model 2 was designed and tested to address the question, “Can EMSs predict psychological symptoms, and do they have an effect on the predictive relationship of trait anger and psychological symptoms?” (Model 2). EMSs were included in the predictive analysis in Model 2. The findings and fit indexes of the model in question are illustrated in Figure 2.

According to the results obtained, when the EMS variable was added to the analysis, significant changes in values were obtained in Model 1. In terms of the model fit indexes, the fit index values obtained in Model 2 were at insufficient levels (*χ*^2^/sd = 2.91; CFI = 0.94; TLI = 0.93; standardized root mean squared residual [SRMR] = 0.062; RMSEA = 061).

Figure 2 presents important findings regarding the mediation of EMSs. According to these findings, first, when the fact that the predictive effect of trait anger on psychological symptoms determined in Model 1 (*β* = 0.28, *p* < 0.01) was considered, significant changes were observed in the predictive coefficients in Model 2 with the inclusion of EMSs in the model, and trait anger predicted psychological symptoms (*β* = 0.13 *p* < 0.01).

**Figure 3:**
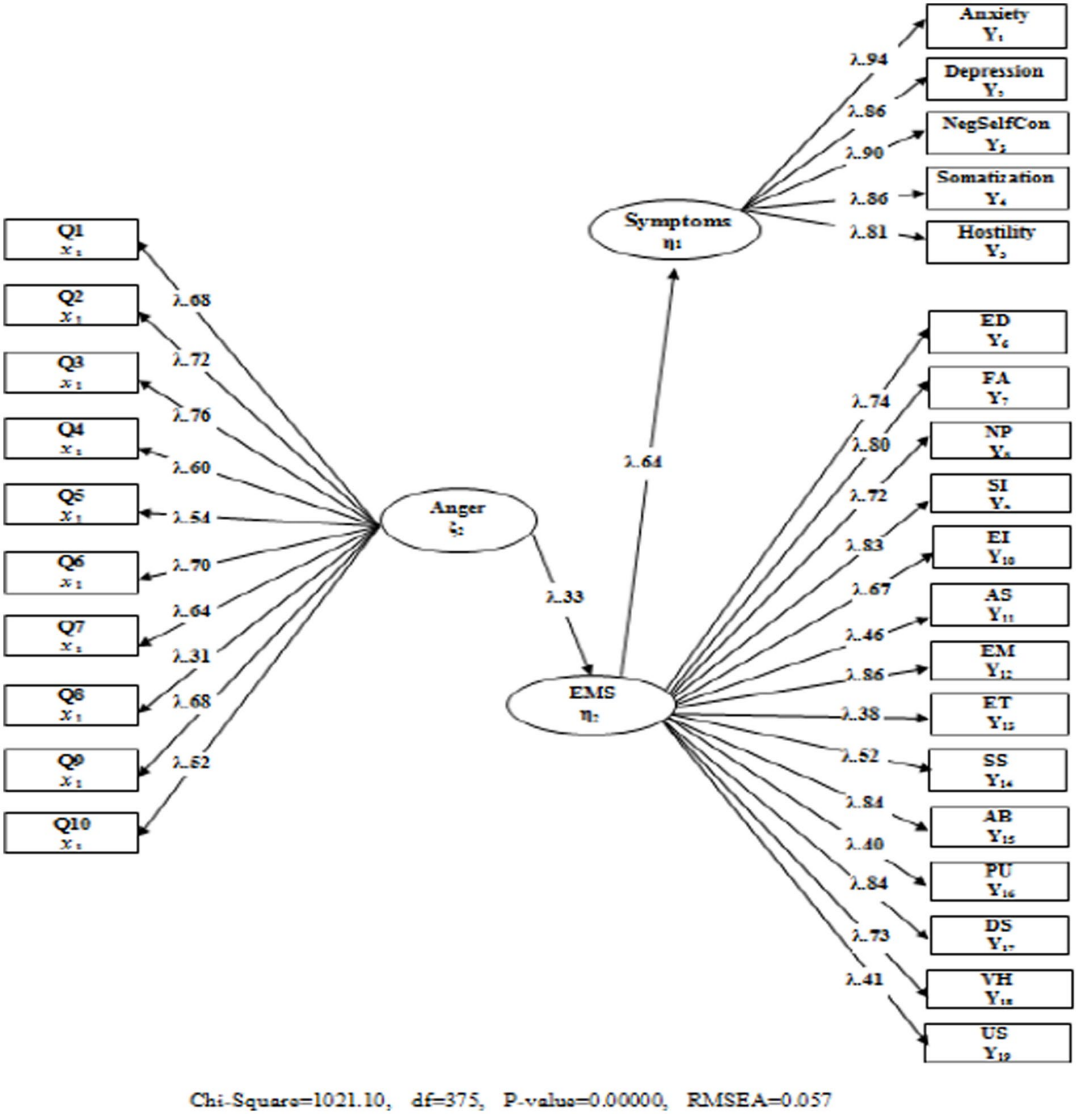
The standardized model regarding the full mediating role of early maladaptive schema.

Two important criteria were discussed to clarify the mediation relationship between the variables. First, the inclusion of the mediator variable in the model (partial mediation) revealed a significant decrease in the predictive coefficient between the two variables, and second, this predictive coefficient became meaningless with the inclusion of the mediating variable in the model (full mediation). In this sense, when the findings obtained from models 1 and 2 were examined, the relationship between trait anger and psychological symptoms was found to be partially mediated by EMSs. In addition, EMSs may have a full mediating effect owing to the significant decrease in the predictive coefficient obtained in Model 2. Accordingly, in the third stage, hypothesis 3 as Model 3 was designed and tested to address the question, “What is the mediating role of EMSs in the relationship between trait anger and psychological symptoms?” The findings obtained are presented in Figure 3.

In Figure 3, the full mediating role of EMSs in the relationship between trait anger and psychological symptoms was examined. The values related to the fit indexes that emerged as a result of the analysis (*χ*^2^/sd = 2.72; CFI = 0.96; TLI = 0.95; SRMR = 0.057; RMSEA = 0.057) indicate that the tested model is valid and that the EMSs had a full mediating role. Considering the predictive coefficients obtained in Model 2, it is understood that there is an improvement in the predictive coefficients of the full mediation model. Accordingly, while trait anger explains 10% (*β* = 0.32, *p* < 0.01) of the variance in the EMSs in Model 2, this rate increased to 11% (*β* = 0.33, *p* < 0.01) in Model 3. While the predictive coefficient of EMSs on psychological symptoms was 27% (*β* = 0.52, *p* < 0.01) in Model 2, this rate was calculated as 41% (*β* = 0.64, *p* < 0.01) in Model 3. On the basis of the positive improvement observed in the predictive coefficients in Model 3, EMSs fully mediate the relationship between trait anger and psychological symptoms.

## 6. Discussion

In line with the main purpose of this study, the mediating role of EMSs, which is the basic concept of schema therapy, was found in the relationship between trait anger and psychological symptoms. The analyses performed for this purpose revealed that at the first stage, trait anger and psychological symptoms, which were the other variables, were positively related to each other. The findings show that trait anger was strongly associated with psychological symptoms. These results answer the question in the first hypothesis of this study and were consistent with the results of previous studies. For example, Nakano and Kitamura (2001) reported high positive correlations between psychological symptoms and anger. Similar studies revealed a statistically positive and significant correlation between anger and various psychological symptoms, especially depression (Turkcapar et al., 2004; Taft et al., 2007; Hisli-Sahin et al., 2011; Oz et al., 2011; Castelli et al., 2013; Leonhardt et al., 2018), posttraumatic stress disorder (Orth et al., 2008; Raab et al., 2013), eating disorder (Zaitsoff et al., 2002), rumination (Christ et al., 2020), and violence (Novaco and Chemtob, 2015).

As the results show, trait anger explained EMSs significantly. Accordingly, the question in the second hypothesis of the study was addressed, and the results were consistent with those of previous studies. Anger was evaluated in relation to its triggering events and was examined in a broad framework in the cognitive-behavioral theory (Deffenbacher, 2011). Studies have shown that these two variables are related. It is remarkable that in the studies examined, the relationship between anger and exposure to neglect and abusive experiences is in the forefront of the formation of EMSs. In another study that investigated the connection between anger and EMSs, emotional bullying and neuroticism were found to be associated with all schema domains. Schemas play a mediating role between bullying and emotional symptoms (Calvete, 2014). In addition, the schemas of exposure to violence and abuse are related to narcissism and anger (Calvete and Orue, 2010).

It is evident that the anger scores of individuals were highly predicted by the abandonment and entitlement schema domains at high levels (Askari, 2018). Among the schema domains of EMSs, disconnectedness, impaired autonomy, and impaired boundaries predicted the difficulty in emotion regulation (Eldoğan and Barışkın, 2014), and anger and perceived maternal control were significantly mediated by the boundary schema (Saritas-Atalar and Altan-Atalay, 2018). A study conducted to examine the impact of EMSs found a positive relationship between the authoritarian attitude of parents and aggression of children, and a negative relationship between the permissive attitude of the mother and the physical aggression of the child (Ikiz and Oztürk, 2016). McKee et al. (2012) concluded in their study that men who experienced trait anger and had a high level of aggression had negative self-schemas. Likewise, men who tended to suppress their anger had negative self-schemas.

From this research, we conclude that EMSs significantly explain psychological symptoms. This result answers the question in the second hypothesis of the study and is consistent with those of other studies. The results obtained from our study should be assessed by considering the fact that “schemas are life-long, widespread, comprehensive, and cognitive characteristics toward one’s self and his/her relationships that predominantly develop during childhood and adolescence” (Andrews et al., 2000; Bagdacicek, 2009; Lee et al., 2015). In addition, the link between EMSs and psychological symptoms has been demonstrated by other studies. For example, some schema domains mediate significant relationships between anxiety and co-rumination (Carlucci et al., 2018).

Related studies have shown that childhood traumas affect depression (McGinn et al., 2005; Hawke and Provencher, 2012), anxiety, and dissociative symptoms and the relationships between these symptoms in different ways (Moscariello et al., 2010). Dysfunctional schemas play a mediating role in the relationship between negative parental attitudes and psychopathology (Hedley et al., 2001; Harris and Curtin, 2002; Seavey and Moore, 2012). In addition, the separation-rejection schema domain plays a mediating role in the relationship between maternal rejection perception and anxiety, and the impaired autonomy-other directedness schema domain plays a mediating role between maternal rejection perception and depression and between maternal rejection perception and anxiety. This study is considered important because it examined the mechanisms that may impact the relationship between perceived maternal rejection and psychological problems, especially anxiety (Saritaş-Atalar and Gençöz, 2015).

The mediating roles of resilience and self-esteem between behavioral and emotional problems have been confirmed by the reports that indicated that psychological abuse in the early life periods has negative relationships with resilience and self-esteem and positive relationships with behavioral and emotional problems (Liu et al., 2014; Arslan, 2016; Arslan and Coşkun, 2020; Arslan and Coşkun, 2022). In another study, a model of the mediating role of self-esteem was tested in the relationship between automatic thoughts and the level of hopelessness. It was concluded that automatic thoughts and self-esteem significantly predict hopelessness (Savi-Çakar, 2014) and have decisive effects on the interpersonal relationship construction style (Çolakoğlu et al., 2015).

The effect of EMSs should not be ignored, especially in the development of personality disorders, which are important actors in problematic interpersonal relations. EMSs play a full mediating role in the relationship between negative childhood experiences and avoidant personality disorders (Carr and Francis, 2010). According to Krause-Utz et al. (2021), borderline personality influences the occurrence of violent behaviors in childhood and later ages. Our findings are consistent with those of other studies that examined the interaction between EMSs and psychological symptoms.

According to the results of the third model, EMSs have a full mediating role in the relationship between trait anger and psychological symptoms. The question in the third hypothesis of the study was answered.

Only few studies have shown the mediating effect of EMSs in the relationship between trait anger and psychological symptoms. Therefore, this finding is considered important. The fact that anger, which is a natural emotion, contributes to psychological symptoms through EMSs indicates that anger is experienced inappropriately. Especially in childhood, children are exposed to inappropriate parenting emotion regulation styles, particularly the ways parents express their anger and aggression. It can be argued that this situation also affects the formation of EMSs in children and leads to an unhealthy development of their emotion regulation skills. The influence of early-life experiences on the formation of personality and personality-related pathologies has been recognized. Thimm (2010a) reported that EMSs and the dimensions of the five-factor personality model, especially neuroticism, overlap significantly.

The results of this study are expected to contribute to the deeper knowledge of researchers, educators, and professionals working with children and parents. On the basis of the results of the study of Orue et al. (2021), changes in socio-cognitive processes in individuals are effective in reducing their aggression and will raise awareness about the regulation of relationships in the family environment. In addition, they present important results for psychologists and psychological counselors in understanding the feeling of anger experienced by individuals. This study shows an important finding in terms of elucidating the role of early experiences in understanding psychological symptoms. It is necessary to demonstrate the consequences that communication with the child in the family and abusive and negligent behaviors will engender in the child’s future life. Emotion regulation skills are acquired from an early age. From this point of view, it is necessary to gain healthy expressions of emotions to individuals from an early age. An appropriate family environment and the role of early life cannot be ignored in achieving this.

### 6.1. Study limitations

The small number of variables used in this study can be considered as a limitation of this study. Anger expression styles should be examined in future studies. Another limitation of this study is that it was conducted with undiagnosed samples. It would be beneficial to repeat the study on a clinical sample. Moreover, the participants were not asked whether they had a psychological disorder and were not screened for this issue. Finally, the cross-sectional research design of this study with the inclusion of a student sample is also a limitation.

## 7. Conclusion

In this study, which was conducted with university students, EMSs were found to play a full mediator role in the relationship between trait anger and psychological symptoms. It is important to take these findings into consideration in psychological counseling studies with young people. Oren and Gençdogan (2007) reported that young individuals need serious psychological help. According to studies aimed at preventing aggression in adolescents, in programs designed to gain anger management skills, it is beneficial to focus on non-functional and automatic thoughts that trigger anger (Yavuzer and Karataş, 2013). Cassiello-Robbins and Barlow (2016) observed that anger is an emotion that has not been studied sufficiently, even though it is important in the development, maintenance, and treatment of emotional disorders. We hope that the results of this study will contribute to treatment studies in the future. Prioritizing EMSs and conducting intervention studies in this direction to modify the non-functional strategies used by individuals to deal with anger will increase treatment success rates. We can argue the same for individuals who have been subjected to traumatic experiences, especially at an early age, and have experienced various psychological problems thereafter.

## Data availability statement

The raw data supporting the conclusions of this article will be made available by the author, without undue reservation.

## Ethics statement

The studies involving human participants were reviewed and approved by Kafkas University Social and Human Sciences Scientific Research and Publication Ethics Committee. The patients/participants provided their written informed consent to participate in this study.

## Author contributions

The author confirms being the sole contributor of this work and has approved it for publication.

## Conflict of interest

The author declares that the research was conducted in the absence of any commercial or financial relationships that could be construed as a potential conflict of interest.

## Publisher’s note

All claims expressed in this article are solely those of the authors and do not necessarily represent those of their affiliated organizations, or those of the publisher, the editors and the reviewers. Any product that may be evaluated in this article, or claim that may be made by its manufacturer, is not guaranteed or endorsed by the publisher.

## Appendix

**Abbreviations in the outputs of Model 2 and 3**

**Table 1:**

ED	Emotional Deprivation
FA	Failure to Achieve
NP	Negativity/Pessimism
SI	Social Isolation/Shame
EI	Emotional Inhibition
AS	Approval-Seeking/Recognition-Seeking
EM	Enmeshment/Undeveloped Self
ET	Entitlement/Grandiosity
SS	Self Sacrifice
AB	Abandonment/Instability
PU	Punitiveness
DS	Defectiveness
VH	Vulnerability to Harm
US	Unrelenting Standards/Hypercriticalness
